# A New Spin on Journal Club: Using Evidence-Based Arguments to Debate Research News

**DOI:** 10.1007/s40670-025-02531-4

**Published:** 2025-11-08

**Authors:** Karie Turley, Terence R. Mitchell, Bonnie Brenseke

**Affiliations:** 1 Campbell University School of Osteopathic Medicine, Lillington, NC USA; 2 Department of Anatomy, Campbell University School of Osteopathic Medicine, Lillington, NC USA; 3 Department of Pathology, Campbell University School of Osteopathic Medicine, Lillington, NC USA

**Keywords:** Journal club, Debate, News

## Abstract

Biomedical master’s students participate in a debate-style journal club that challenges them to evaluate a health-related finding reported in the news. Assigned opposing views, students must defend their position using evidence from the scientific literature. This approach to journal club mirrors real-life situations and has been positively received by students.

In recent years, news reports have been increasingly questioned and accused of being false or fake. Science has been doubted and disputed. Healthcare professionals are often asked to support or refute public claims. For pre-health students, staying current with scientific literature and effectively communicating evidence-based research are essential skills.

Journal club, a longstanding component of medical education, traditionally operates as facilitator-guided group discussions of an article [1–3] . In 2019, faculty at Campbell University School of Osteopathic Medicine (CUSOM) developed a debate-style journal club where students read a news report on a research study. In this debate-style format, students are assigned to either the affirmative or negative position on the topic presented in the news report. The groups then find and present evidence from peer-reviewed journals to support their stance or challenge the opposing view.

At CUSOM, journal club is part of the Master of Science in Biomedical Sciences program. In the first year, students follow the traditional format, presenting and discussing assigned articles. In the second year, now experienced with article interpretation and presentation, students transition to the debate-style format. Course objectives include analyzing data to make evidence-based interpretations and evaluating strengths and weaknesses of scientific literature. The course has six two-hour debate sessions plus an “Introduction to the Course” and “End-of-Course Debrief & Peer Feedback.” During the course introduction, students receive group assignments, news reports, debate positions, and debate format instructions. Debates involve four student groups (3–6 students/group)—two debating and two serving as the audience.

On debate day, the debating groups submit an opening argument and figures from journal articles validating their position. Audience groups submit a summary of the debate afterward.

Each session opens with every student taking a five-question quiz on the news report. The debate structure is as follows:

- Opening argument (affirmative, then negative): 5 min each
- Group work: 15 min
- Presentation of evidence (two figures from affirmative, then negative): 3 min to explain figure and 7 min for open discussion, thus 10 min per figure/20 min each
- Group work: 15 min
- Closing argument (affirmative, then negative): 5 min each
- Debriefing and decision on “most persuasive”: 10 min
- Total debate time is 100 min; 20-min buffer time is available

The instructor moderates the session. During group work time, debaters strategize responses, choose what evidence best supports their position, and work on their closing argument, while audience members summarize what was presented, develop questions to ask during times for discussion, and establish which group they think is most persuasive and why. At the end of each session, the instructor leads a debrief and announces the “most persuasive” group. At the end of the academic term, the group selected the most receives special recognition.

Each year, the course directors review the news reports and associated debate positions. Based on student feedback and other considerations, some debate topics are revised or replaced. News reports are pulled from various outlets (e.g., NBC, ScienceDaily) and headline a health claim drawn from a research study (e.g., drinking coffee helps you live longer, E-cigarettes help people quit smoking). Over the years, debate positions have included high adoption rates of mask use lower (vs. do not lower) the prevalence of respiratory disease within a population and moderate alcohol consumption benefits (vs. having negative or neutral impacts on) human health.

Survey data from 2019 to 2024 show that most students viewed the course favorably (Table 1). Students commented positively on the activeness of the sessions, describing them as “fun” and “engaging.” A consistent concern was that certain debate positions lacked sufficient support from the scientific literature, which contributed to a perception of one-sidedness. Despite this, students reported gaining confidence in evaluating scientific literature. For instance, a student commented, “I learned a lot, and gained a lot of confidence in my ability to work through a complex issue,” while another shared, “My area of critical thinking from a research perspective started with this course.” Student comments also reflected growth in professional skills, including teamwork, respectful discourse, and constructive feedback. These findings indicate that teaching pre-health students to debate research news using evidence-based arguments offers an engaging alternative to traditional journal club and may enhance public and patient communication.

**Table 1:** Student level of agreement with survey question

Survey question	Agree	Undecided	Disagree
The organization and structure of this course facilitated my learning	97.1%	1.4%	1.4%
The course has helped me develop critical thinking skills that can be applied to clinical practice or research	97.1%	1.4%	1.4%
The overall quality and value of this course was excellent	94.3%	2.9%	2.9%

A Likert scale was part of the course evaluation with seven options for respondents to express their level of agreement or disagreement with the statement. For ease of reporting, the options of Agree Somewhat, Agree, and Strongly Agree were combined to create the ‘Agree’ column reported in the table. Similarly, for ‘Disagree’, the options of Disagree Somewhat, Disagree, and Strongly Disagree were combined. Data reflect 70 student evaluations collected from 2019–2024

## Data Availability

Anonymized student evaluation data supporting the findings of this study are available from the corresponding author upon reasonable request and subject to institutional approval.
